# Comparing biological effectiveness guided plan optimization strategies for cranial proton therapy: potential and challenges

**DOI:** 10.1186/s13014-022-02143-x

**Published:** 2022-10-22

**Authors:** Christian Hahn, Lena Heuchel, Jakob Ödén, Erik Traneus, Jörg Wulff, Sandija Plaude, Beate Timmermann, Christian Bäumer, Armin Lühr

**Affiliations:** 1 Department of Physics, TU Dortmund University, Dortmund, Germany; 2 OncoRay – National Center for Radiation Research in Oncology, Faculty of Medicine and University Hospital Carl Gustav Carus, Technische Universität Dresden, Helmholtz-Zentrum Dresden-Rossendorf, Dresden, Germany; 3 Department of Radiotherapy and Radiation Oncology, Faculty of Medicine and University Hospital Carl Gustav Carus, Technische Universität Dresden, Dresden, Germany; 4 RaySearch Laboratories AB, Stockholm, Sweden; 5 West German Proton Therapy Centre Essen, Essen, Germany; 6 West German Cancer Center (WTZ), University Hospital Essen, Essen, Germany; 7 Department of Particle Therapy, University Hospital Essen, Essen, Germany; 8 German Cancer Consortium (DKTK) and German Cancer Research Center (DKFZ), Heidelberg, Germany

**Keywords:** Proton therapy, Treatment plan optimization, Linear energy transfer (LET), Relative biological effectiveness (RBE), Track ends, Dirty dose

## Abstract

**Background:**

To introduce and compare multiple biological effectiveness guided (BG) proton plan optimization strategies minimizing variable relative biological effectiveness (RBE) induced dose burden in organs at risk (OAR) while maintaining plan quality with a constant RBE.

**Methods:**

Dose-optimized (DOSEopt) proton pencil beam scanning reference treatment plans were generated for ten cranial patients with prescription doses ≥ 54 Gy(RBE) and ≥ 1 OAR close to the clinical target volume (CTV). For each patient, four additional BG plans were created. BG objectives minimized either proton track-ends, dose-averaged linear energy transfer (LET_d_), energy depositions from high-LET protons or variable RBE-weighted dose (D_RBE_) in adjacent serially structured OARs. Plan quality (RBE = 1.1) was assessed by CTV dose coverage and robustness (2 mm setup, 3.5% density), dose homogeneity and conformity in the planning target volumes and adherence to OAR tolerance doses. LET_d_, D_RBE_ (Wedenberg model, α/β_CTV_ = 10 Gy, α/β_OAR_ = 2 Gy) and resulting normal tissue complication probabilities (NTCPs) for blindness and brainstem necrosis were derived. Differences between DOSEopt and BG optimized plans were assessed and statistically tested (Wilcoxon signed rank, α = 0.05).

**Results:**

All plans were clinically acceptable. DOSEopt and BG optimized plans were comparable in target volume coverage, homogeneity and conformity. For recalculated D_RBE_ in all patients, all BG plans significantly reduced near-maximum D_RBE_ to critical OARs with differences up to 8.2 Gy(RBE) (*p* < 0.05). Direct D_RBE_ optimization primarily reduced absorbed dose in OARs (average ΔD_mean_ = 2.0 Gy; average ΔLET_d,mean_ = 0.1 keV/µm), while the other strategies reduced LET_d_ (average ΔD_mean_ < 0.3 Gy; average ΔLET_d,mean_ = 0.5 keV/µm). LET-optimizing strategies were more robust against range and setup uncertaintes for high-dose CTVs than D_RBE_ optimization. All BG strategies reduced NTCP for brainstem necrosis and blindness on average by 47% with average and maximum reductions of 5.4 and 18.4 percentage points, respectively.

**Conclusions:**

All BG strategies reduced variable RBE-induced NTCPs to OARs. Reducing LET_d_ in high-dose voxels may be favourable due to its adherence to current dose reporting and maintenance of clinical plan quality and the availability of reported LET_d_ and dose levels from clinical toxicity reports after cranial proton therapy. These optimization strategies beyond dose may be a first step towards safely translating variable RBE optimization in the clinics.

## Background

In proton therapy treatment planning and delivery, a generic constant relative biological effectiveness (RBE) of 1.1 is used to account for the higher cell killing efficiency of protons over photons [1, 2]. However, in-vitro and in-vivo studies show that RBE varies within a treatment field as a function of physical and biological parameters [3, 4]. While emerging clinical studies showed that RBE varies with the linear energy transfer (LET) and tissue-specific radiosensitivity [5–11], some studies did not find correlations between LET and toxicity with the interpatient variability being a potential obscuring effect [12, 13]. The uncertainty in RBE already affects daily clinical proton therapy treatment planning strategies [14, 15] and urges the exploration and development of treatment planning strategies beyond absorbed dose optimization [16, 17].

LET and RBE increase as protons slow down and reach their maximum at the end of beam range [3]. Since the distal edge of the treatment field is placed in normal tissue, RBE variability may increase the risk for normal tissue toxicity in adjacent organs at risk (OAR) [18]. Recent studies on brain tumor patients reported that radiation-induced image changes occur at the distal edge of proton treatment fields, where high absorbed dose coincides with elevated LET and RBE [5, 7, 8, 10]. Therefore, proton therapy centers avoid fields stopping in front or within OARs, avoid acute opening angles between incident beams or add more fields to reduce the relative weight of inevitable beams stopping in OAR proximity [14, 15]. However, a recent replanning study for glioma patients concluded that these clinical strategies are only effective for some patients and biologically guided treatment planning strategies beyond absorbed dose are needed for an effective risk reduction in normal tissues in view of a variable RBE [19].

Dosimetrically equivalent treatment plans can feature different underlying LET distributions in the same anatomical region [20]. This allows for maintaining the use of a constant RBE in the target volume to ensure tumor control, as recently recommended [2], while reducing LET and variable RBE induced dose burden in normal tissues through optimization. Hence, treatment plans with more favorable LET and, thus, variable RBE distributions in healthy tissue can be created through biologically guided treatment planning strategies without compromising target volume coverage. Optimization approaches beyond absorbed dose were studied individually and include the optimization of LET [21–25], variable RBE with different in-vitro data based models [26, 27] or the redistribution of proton track-ends, which spatially coincide with high LET and absorbed dose [28]. While different groups demonstrated these biological effectiveness guided optimization techniques to be effective on their own, strengths and weaknesses of each strategy will only become visible in a direct comparison and must be evaluated before considering translation of these approaches into clinical studies.

This study introduces and compares four optimization strategies beyond absorbed dose optimization to mitigate variable RBE-induced dose burden in OARs while ensuring target dose coverage and plan quality with a constant RBE. All treatment plans were designed in line with today’s clinical practice and utilized the clinical treatment field angles used for that patient. Planning strategies include the optimization of track-end distributions, dose-averaged LET (LET_d_) in voxels of high absorbed dose, variable RBE-weighted dose (D_RBE_) and the introduction of a new approach penalizing dose contributions by high-LET protons (‘dirty dose’). The potential of each optimization strategy to reduce LET_d_, D_RBE_ and normal tissue complication probability (NTCP) in OARs is assessed for ten cranial proton therapy patients. This study aims towards safely translating optimization with variable RBE into clinical practice.

## Methods

### Patients and general treatment planning

All patients included in this analysis were covered by the ethics approval of a prospective registry study (German Clinical Trial Register: DRKS00004384) and provided written informed consent. Ten cranial proton therapy patients were selected with prescription doses of 54 Gy(RBE) or higher, using an RBE of 1.1, and one or more dose-limiting serial OAR close to the clinical target volume (CTV, Table 1). Treatment planning was based on the planning target volumes (PTV), defined as 5 mm isotropic expansion of the CTV to account for range and setup uncertainties of 3.5% and 2 mm, respectively. Multi-field optimized (MFO) proton pencil beam scanning treatment plans were created using the clinical beam model from the IBA Proteus Plus (IBA PT, Louvain-la-Neuve, Belgium) dedicated nozzle at The West German Proton Therapy Centre Essen and the research treatment planning system (TPS) RayStation v8.99.30.101 (RaySearch Laboratories AB, Stockholm, Sweden). Treatment plans were deemed clinically acceptable if their constant RBE-weighted dose distribution (D_1.1_) did not exceed OAR tolerance doses and showed adequate CTV coverage. CTV underdosages were only accepted to fulfil OAR tolerance doses. Clinically acceptable reference treatment plans were created using solely objectives for the absorbed dose multiplied with a constant RBE of 1.1 (D_1.1_) and are hereafter termed DOSEopt plans. Simultaneous integrated boost concept was used for patients with two CTVs (Table 1). A grid size of 2 × 2 x 2 mm^3^ and a statistical Monte Carlo (MC) uncertainty of 0.5%, defined as the arithmetic mean of one standard deviation in dose across all voxels with doses higher than 50% of a beam’s maximum dose, for the final dose calculation were used. For each patient, the same and clinically used treatment field angles were used for DOSEopt and for the biological effectiveness guided (BG) treatment plans, respectively (Table 1).

**Table 1:** Patient and treatment planning characteristics of all patients

Patient number	Diagnosis	Prescribed median dose/Gy(RBE)		Fx	Dose-limiting OARs	Treatment field angles (Gantry, Couch)
		Primary CTV	Secondary CTV			
1	Meningioma	54	–	30	Brainstem	(90°,310°) (120°,0°)^a^ (70°,0°)^a^
2	Rosette-forming glioneuronal tumor, relapse	54	–	30	Opt. L + R, Chiasm, Brainstem	(90°,350°)^a^ (90°,190°)^a^ (90°,270°)
3	Meningioma	54	–	30	Opt. L + R, Chiasm, Brainstem	(90°,350°)^a^ (90°,320°)^a^ (90°,180°)
4	Meningioma	54	–	30	Opt. L, Chiasm, Brainstem	(90°,0°)^a^ (90°,320°)^a^ (90°,270°)
5	Meningioma	54	–	30	Opt. L + R, Chiasm, Brainstem	(290°,0°)^a^ (240°,0°)^a^ (90°,0°)
6	Chondrosarcoma	69.3	54.45	33	Opt. L + R, Chiasm, Brainstem	(90°,190°)^a^ (90°,350°)^a^ (90°,250°)
7	Adenoidcystic carcinoma	70	63	35	Opt. L, Chiasm, Brainstem	(240°,10°)^a^ (25°,0°)^a^ (105°,0°)
8	Clivus chordoma	73.5	56	35	Opt. L + R, Chiasm, Brainstem	(60°,0°)^a^ (120°,0°)^a^ (270°,0°)^a^
9	Chordoma	73.5	56	35	Opt. L + R, Chiasm, Brainstem	(100°,355°)^a^ (55°,290°)^a^ (240°,0°)
10	Clivus chordoma	73.5	56	35	Opt. L + R, Chiasm, Brainstem	(100°,0°)^a^ (250°, 5°^)a^ (60°,0°)^a^

*CTV* clinical target volume, *Fx* fractions, *OAR* organ at risk, *Opt* optic nerve, *L* left, *R* right, a: with rangeshifter

### Biological effectiveness guided treatment plan optimization

**Fig. 1:**
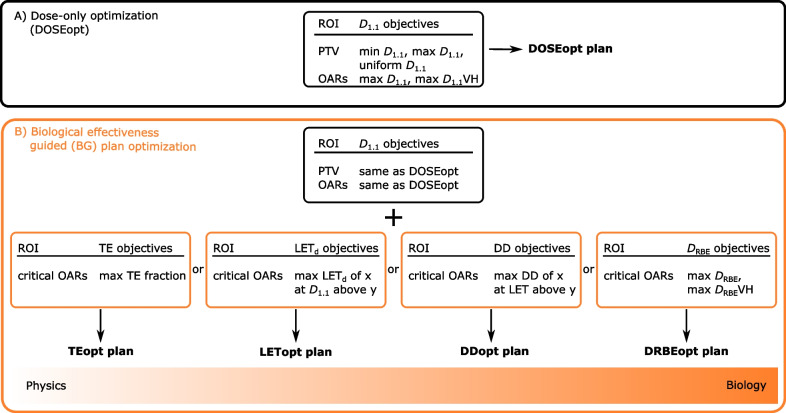
Objectives and workflow to create treatment plans based on dose-only and biological effectiveness guided optimization with the aim to reduce variable relative biological effectiveness (RBE) weighted dose (D_R__B__E_) to critical organs at risk (OAR) while maintaining target coverage, robustness, dose homogeneity and conformity. **A**) Clinically acceptable reference plan using only objectives for absorbed dose**,** thus D_1.1_ (DOSEopt). **B**) Maintaining the same DOSEopt objectives and adding objectives for one of the four different biological effectiveness guided (BG) optimization strategies. Abbreviations: ROI: region of interest, DVH: dose-volume histogram, PTV: planning target volume, TE: track-ends, LET_d_: dose-averaged linear energy transfer, DD: dirty dose, D_RBE_: variable RBE-weighted dose, opt: optimization

This study aimed to reduce the variable RBE-weighted dose (D_RBE_) in dose-limiting serial OARs, while keeping the absorbed dose, thus D_1.1_, to the CTV comparable to the corresponding DOSEopt reference plan. First, the weights and levels of the D_1.1_-based objectives found with DOSEopt were transferred without modification to the optimization of the BG treatment plans. Second, for each BG optimization (BGopt) planning strategy, specific objective functions beyond D_1.1_ were added to reduce D_RBE_ to dose-limiting serial OARs, hereafter termed critical OARs (Fig. 1, Table 1). To allow for a D_RBE_ reduction through a redistribution of stopping protons, a small increase in mean D_1.1_ to healthy brain tissue is expected. In accordance with similar previous studies, this increase was kept below 3% [22, 28]. The four different BG optimization strategies ranged from physical to biological optimization approaches of variable RBE-driving factors and each approach covered the optimization of one of the following quantities:

- proton track-end distributions (TEopt),
- proton dose-averaged LET (LET_d_) in voxels above a dose threshold (LETopt),
- dose contribution by high-LET protons, i.e. dirty dose (DDopt) and
- variable RBE-weighted dose (DRBEopt).

Hence, four BG treatment plans were created for each patient.

Proton track-ends are defined as the location where protons stop, i.e. the voxel where the proton transport is terminated. TEopt exploits that the maximum of the proton track-end depth distribution spatially correlates with the absorbed dose maximum and elevated LET_d_ area. In TEopt, the track-end fraction, defined as the number of track-ends in the region of interest divided by the total number of track-ends in the patient, was minimized within the region of interest (ROI). TEopt objectives acted on primary protons and aimed to reduce the track-end fraction in the critical OAR. Here, a reduction of track-end fractions in critical OARs by 50% relative to those in the corresponding DOSEopt plan was applied to all patients.

LETopt minimizes the LET_d_, which is a dose-weighted mean value of the LET-spectrum within a voxel. LET_d_ is a measure for radiation quality and requires a sensible weighting with local dose to effectively reduce D_RBE_, since the highest LET_d_ values usually occur in volumes of negligible dose and are biologically irrelevant. Therefore in LETopt, LET_d_ values above a set LET_d_ level were only penalized in voxels with a dose above a user-specified threshold. Clinical follow-up studies consistently reported LET_d_ values around 2.5 keV/µm and D_1.1_ above 40 Gy(RBE) in radiation-induced image change areas after cranial proton therapy [8, 12, 13, 29–31]. Accordingly, here, LET_d_ values above 2.5 keV/µm were penalized in critical OAR but only in voxels with D_1.1_ above 40 Gy(RBE). LET_d_ was defined as the unrestricted dose-averaged LET for all protons to unit density tissue and calculated with Method ‘C’ from [32].

In the ‘dirty dose’ approach, the DDopt objective is designed to penalize energy depositions from protons with an LET above a set LET threshold. The absorbed total dose in each voxel was separated in two categories: the absorbed dose deposited by individual protons with LET above the LET threshold (‘dirty dose’) and the absorbed dose deposited by individual protons below the LET threshold (‘clean dose’). Typically, the dirty dose portions of each treatment field are located at the distal field edge. DDopt is a dose-optimization approach where only the dirty dose portion, contributed by high LET protons, is minimized. In this work, the absorbed dose contributed by protons with an LET above 2.5 keV/µm in critical OARs was considered as ‘dirty dose’ and reduced in critical OARs. A reduction of near-maximum dirty dose in critical OARs by 50% relative to those in the corresponding DOSEopt plan was applied to all patients.

DRBEopt optimizes the product of total absorbed dose and variable RBE. Thus, absorbed dose or variable RBE or both can be optimized in order to lower the D_RBE_ in the OAR. DRBEopt was based on the variable RBE model from Wedenberg et al. [33] with α/β = 2 Gy in the critical OARs and the proton absorbed dose and LET_d_ as input parameters. Objective RBE-weighted dose levels applied in DRBEopt with a variable RBE were identical to the D_1.1_ levels applied in the corresponding DOSEopt plan.

During BGopt, track-end fractions, LET_d_, dirty dose or D_RBE_ were scored voxel-wise and were consequently minimized within the critical OAR of the patient. Each of the BG objectives was added as quadratic penalty function to the standard composite objective function in the research TPS for critical OARs (Table 1). All BGopt objectives were implemented as maximum objectives meaning that a penalty is applied when a certain quantity is above a user-specified threshold value. All resulting DOSEopt and BGopt plans are clinically deliverable.

### Plan evaluation

CTV coverage was defined relative to the prescribed dose, using a constant RBE, with D_50%_ = 100% and D_95%_ > 95%, where DX% represents the dose in X% of the CTV. Accordingly, near-maximum and near-minimum doses were defined as the D_1.1_ to 1% and 99% of the volume of interest, respectively. For patients receiving 54 Gy(RBE) to the primary CTV (Table 1), tolerance doses to the brainstem and optical apparatus were 54 Gy(RBE) [34, 35]. For patients with prescription doses > 54 Gy(RBE) to the primary CTV, doses up to 60 Gy(RBE) were tolerated in the optical structures [35]. Similarly for these patients, higher brainstem doses were tolerated if the dose to the brainstem core, defined as a 4 mm circular ROI in the geometric center of the brainstem, did not exceed 54 Gy(RBE) [36]. All OARs were assumed free of tumor cells. Doses to other healthy tissue were kept as low as reasonably achievable and in line with the report on quantitative analyses of normal tissue effects in the clinic (QUANTEC) [37].

Treatment plan quality was defined by dose coverage (D_95%_ > 95%) and robustness in the CTV, dose conformity and dose homogeneity in the PTV as well as adherence to OAR tolerance dose. It was assessed for all DOSEopt and BGopt plans based on their respective D_1.1_ distributions. Dose conformity index (CI) and dose heterogeneity index (HI) for D_1.1_ were defined as,

1 $${\text{CI}} = {\text{V}}_{{{\text{PTV}}}} \left( {{\text{covered}}\;{\text{by}}\;{95}\% \;{\text{isodose}}} \right)/{\text{V}}_{{{95}\% \, \text{isodose} }}$$

2 $${\text{HI}} = \left( {{\text{D}}_{{{2}\% }} /{\text{D}}_{{{98}\% }} } \right)_{{{\text{PTV}}}}$$

with V being the volume of interest. Robustness analysis based on D_1.1_ distributions was performed for all DOSEopt and BGopt plans with twelve scenarios for each treatment plan considering ± 3.5% density uncertainty and isotropic setup uncertainty of ± 2 mm [38] along the cardinal directions in the patient coordinate system [39], for which the percentage of scenarios passing D_95%_ > 95% in the CTVs was determined.

MC scoring extensions of the research TPS were used to recalculate the track-ends, LET_d_ and D_RBE_ on voxel-level for all treatment plans. LET_d_ was only evaluated in voxels with a minimum total absorbed dose of 2 Gy. For D_RBE_ recalculations of the treatment plans, the Wedenberg RBE model was applied with α/β = 10 Gy in the CTVs, 2 Gy otherwise [36] and proton LET_d_ as input parameters. The relative seriality model [40] was used to estimate NTCP for D_RBE_ distributions with equivalent doses delivered in 2 Gy fractions using an α/β of 2 Gy. NTCP values were determined for brainstem necrosis and blindness in the optical structures using D_RBE_ and model parameters of relative seriality s = 1, slope γ = 2.4, D_50_ = 65.1 Gy(RBE) and s = 1, γ = 2.5, D_50_ = 65.0 Gy(RBE), respectively [41], and denoted as NTCP(D_RBE_). Intra-patient differences in D_1.1_, LET_d_, D_RBE_ and NTCP(D_RBE_) between DOSEopt and BGopt plans were derived and statistically tested with a Wilcoxon signed-rank test on a significance level of 0.05.

## Results

**Fig. 2:**
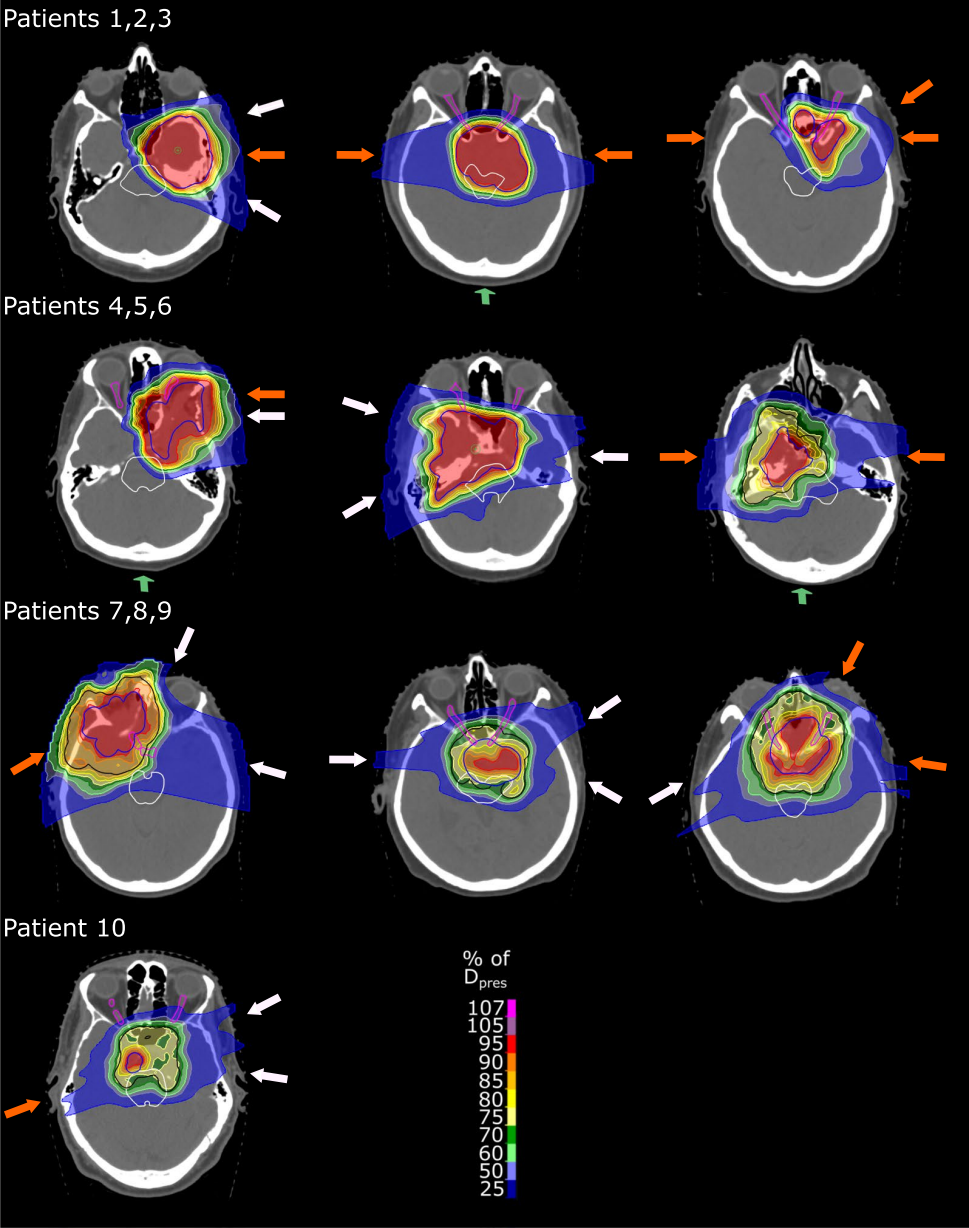
Constant relative biological effectiveness weighted dose distributions of the dose-only optimized reference plans in representative computed tomography slices. Isodoses are displayed relative to the prescription dose to the primary (high-dose) clinical target volume. Arrows depict coplanar (white) and non-coplanar beams (orange) as well as beams passing through the skullcap (green), respectively. Contours show the primary (high-dose) planning target volume (PTV, blue), secondary PTV (black), brainstem (white) and both optic nerves and chiasm (all magenta)

**Fig. 3:**
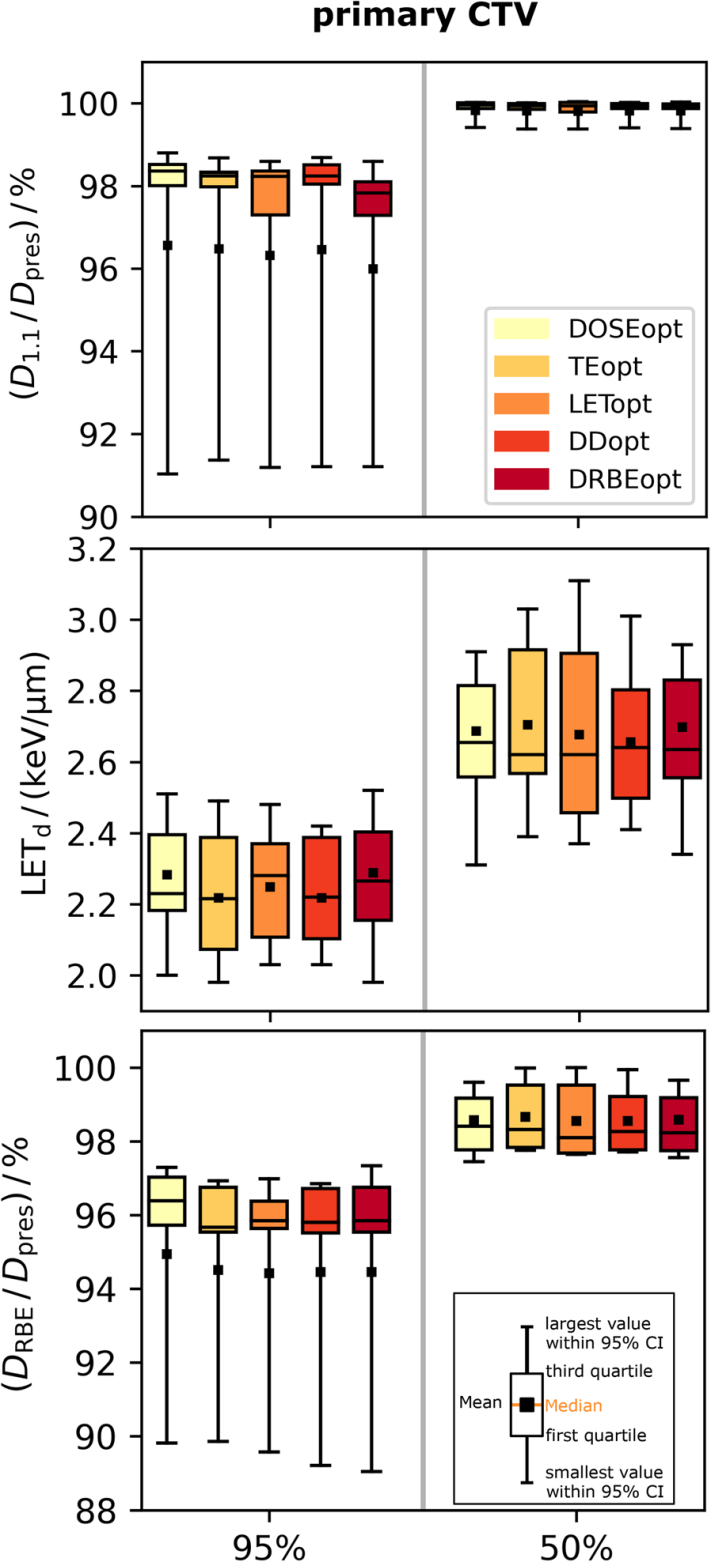
Each boxplot contains near-minimum (95%) or median (50%) values of all patients in the primary clinical target volume (CTV). Constant (D_1.1_, first row) and variable RBE-weighted dose (D_RBE_, third row) volume-histogram values were normalized to the respective prescription dose (D_pres_). Dose-averaged linear energy transfer (LET_d_, second row) is displayed in absolute values. Abbreviations: DOSEopt: dose-only optimization, TEopt: track-end optimization, LETopt: dose-averaged linear energy transfer (LET_d_) optimization with dose threshold, DDopt: Dirty dose optimization, DRBEopt: variable RBE-weighted dose optimization

Using a constant RBE of 1.1, CTV coverage was acceptable for all 50 patient treatment plans with an average (range) median dose to the primary CTV of 99.8 (99.1–100.1)% and D_95%_ to the primary and secondary CTVs of 96.4 (84.9–99.0)% and 99.6 (99.4–102.8)%, respectively (Fig. 2, Fig. 3). PTV coverage was homogeneous (HI: 0.87 (0.73–0.95)) and conformal (CI 0.91 (0.78–1.0)). Intra-patient differences in median dose, D_95%_, CI and HI between BGopt and DOSEopt plans were small with 0.02 (0.00–0.07) Gy(RBE), 0.2 (0.0–1.4) Gy(RBE), 0.01 (0.00–0.07) and 0.01 (0.00–0.04), respectively. For all treatment plans and both prescription groups, near-maximum D_1.1_ values in the brainstem, brainstem core and optical apparatus were acceptable. Increases in mean D_1.1_ to healthy brain tissue were below 3.0% for all BGopt plans. For patients 8 and 10, D_95%_ < 95% and thus decreased CTV robustness as well as HI < 0.8 to the primary CTV were accepted in all plans to fulfill tolerance doses of overlapping critical OARs (Fig. 2).

For on average (range) 96 (67–100)% of uncertainty scenarios, DOSEopt plans fulfilled D_95%_ > 95% to the CTVs. BGopt plans were comparably robust with on average 94 (75–100)% acceptable scenarios. Within each patient, robustness of DOSEopt and BGopt plans were comparable with generally more than 80% of uncertainty scenarios reaching D_95%_ > 95%. While all BGopt strategies were similarly robust for patients with 54 Gy(RBE) prescription doses (on average 93% to 96% robust scenarios), DRBEopt appeared slightly but systematically less robust compared to the other strategies in patients with higher prescribed doses (on average 92% with DRBEopt vs. 97% to 100% robust scenarios with TEopt, LETopt and DDopt).

For D_RBE_ in the CTV, DOSEopt and BGopt plans showed similar absorbed dose and LET_d_ distributions and remained comparable with average D_RBE_ differences in D_95%_ and D_50%_ below 0.3 Gy(RBE) (Fig. 3). Using a variable RBE for dose evaluation, near-maximum D_RBE_ to each critical OAR increased on average by 6.9–8.6 Gy(RBE) for DOSEopt plans and exceeded their respective tolerance doses, i.e. brainstem (9/10 cases), chiasm (9/9), right optic nerve (6/7) and left optic nerve (8/9). Accordingly, NTCP(D_RBE_) for brainstem necrosis and blindness in the chiasm, right and left optic nerve in the DOSEopt plans were 6.6 (0.7–18.0)%, 16.8 (11.0–22.0)%, 9.7 (0.2–20.0)% and 11.2 (1.0–30.0)%, respectively.

**Fig. 4:**
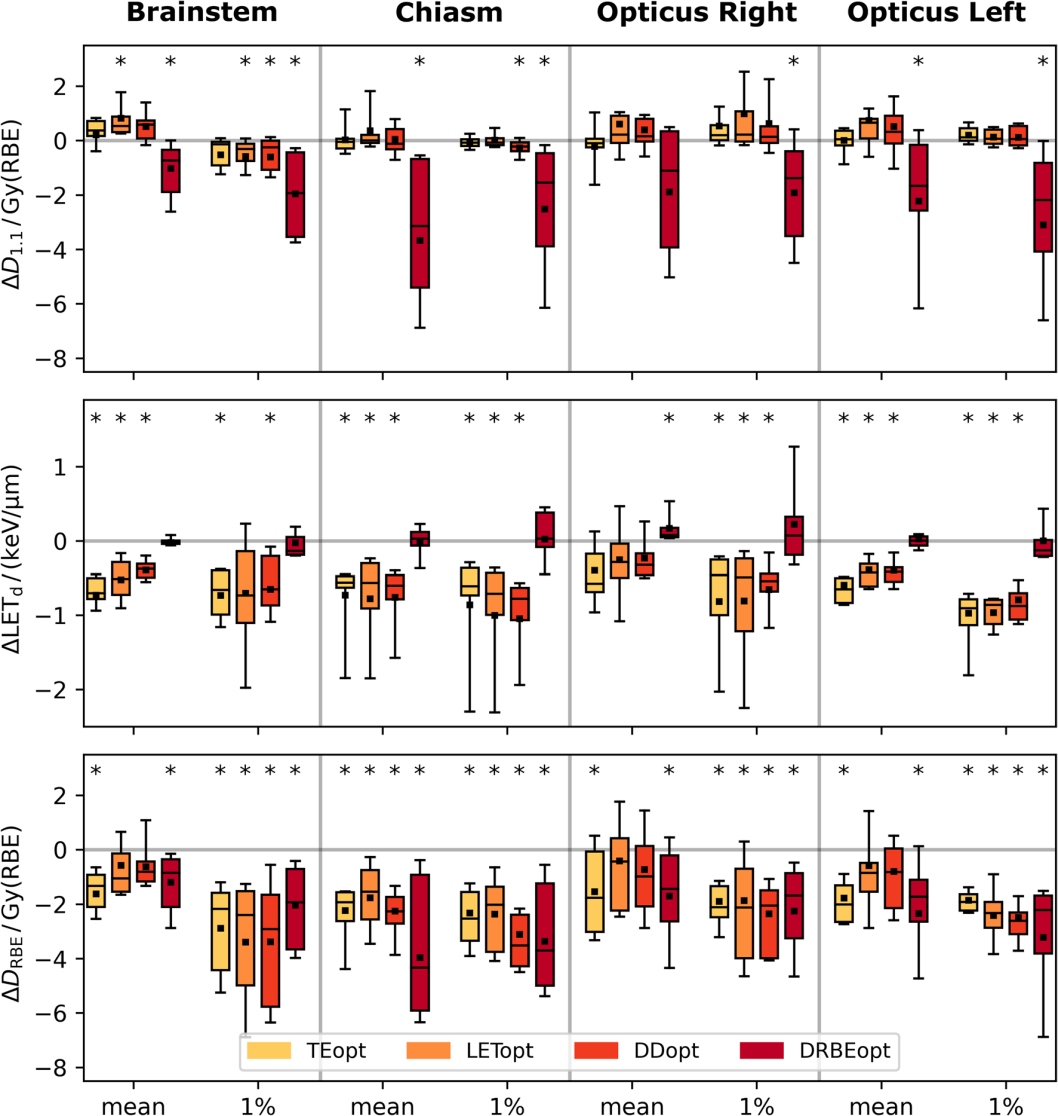
Each boxplot contains differences in mean or near-maximum (1%) values between biological effectiveness guided plans and dose-only optimized plans in critical organs at risk for all patients. Absolute differences in constant (D_1.1_, first row) and variable RBE-weighted dose (D_RBE_, third row) and dose-averaged linear energy transfer (LET_d_, second row) are shown. Statistically significant differences (*p* < 0.05) are marked with an arterisk. Abbreviations: TEopt: track-end optimization, LETopt: dose-averaged linear energy transfer (LET_d_) optimization with dose threshold, DDopt: Dirty dose optimization, DRBEopt: variable RBE-weighted dose optimization

All BGopt plans showed significantly lower near-maximum D_RBE_ to all critical OARs with reductions up to 8.3 Gy(RBE) (Fig. 4). BG optimization functions decreased D_RBE_ to critical OARs by altering absorbed dose, LET_d_ or both. TEopt, LETopt and DDopt did not significantly reduce mean absorbed doses to any critical OAR compared to DOSEopt and differences to DOSEopt were on average below 0.3 Gy (*p* > 0.05, Fig. 4). These three physical strategies reduced mean and near-maximum LET_d_ on average by 0.5 keV/µm and 0.8 keV/µm, respectively, which in both LET_d_ parameters was significantly lower than DOSEopt for three of four critical OARs. In contrast, the DRBEopt approach primarily reduced the mean and near-maximum absorbed dose to critical OARs by on average 2.0 Gy and 2.2 Gy, respectively. Accordingly, these absorbed dose values were significantly lower than for DOSEopt for all but one comparison. No significant LET_d_ reduction was observed for DRBEopt and minor changes in mean and near-maximum LET_d_ and average differences remained below 0.1 keV/µm.

**Fig. 5:**
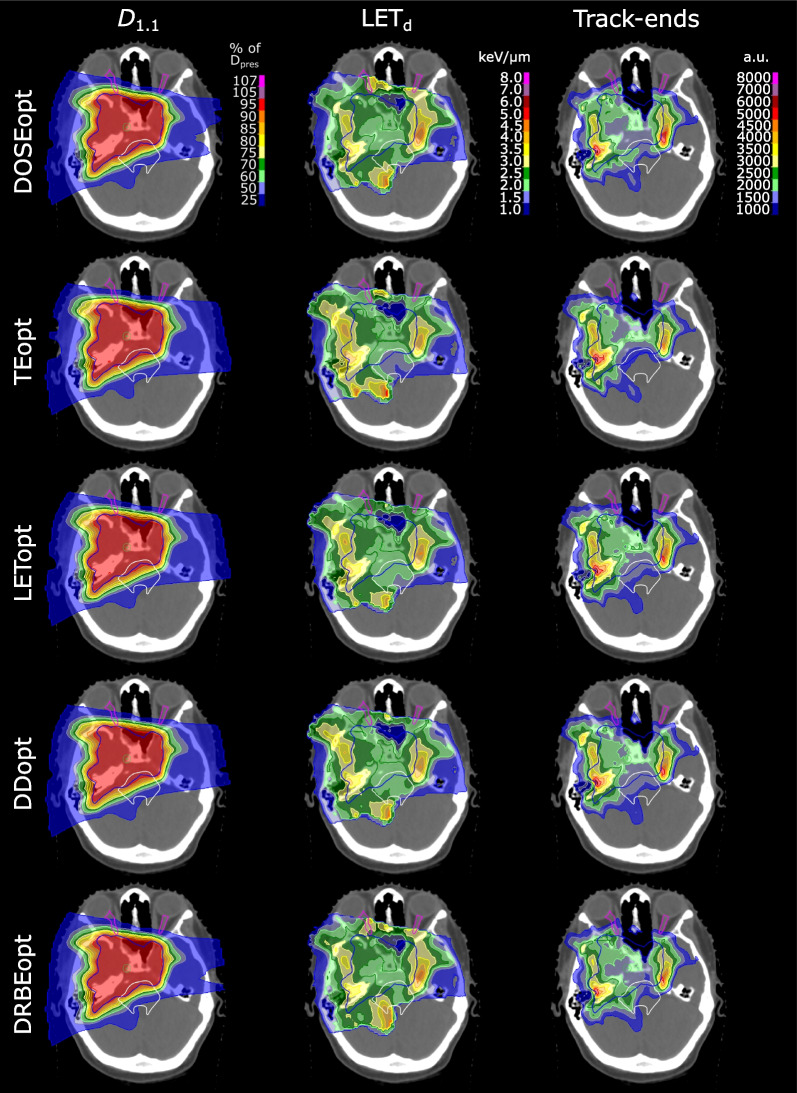
Patient 5: Columns display distributions of constant relative biological effectiveness (RBE) weighted dose (*D*_*1.1*_), dose-averaged linear energy transfer (LET_d_) in voxels above 10 Gy and proton track-ends. Each row shows *D*_*1.1*_, LET_d_ and track-end distributions for one optimization strategy. Primary planning target volume (blue) is shown together with the brainstem (white) and both optic nerves (magenta). DOSEopt: dose-only optimization, TEopt: track-end optimization, LETopt: dose-averaged linear energy transfer (LET_d_) optimization with dose threshold, DDopt: Dirty dose optimization, DRBEopt: variable RBE-weighted dose optimization

LET_d_ reductions were realized by a redistribution of track-ends to areas outside of the critical OARs, as exemplarily shown for patient 5 (Fig. 5). For the DOSEopt plan, many track-ends were placed at the border between PTV and critical OARs to produce the steep absorbed dose gradient necessary to fulfill the respective OAR tolerance doses. This caused areas of LET_d_ ≥ 2.5 keV/µm in and around the critical OARs. TEopt, LETopt and DDopt redistributed track-ends to the PTV and healthy brain tissue, which were not subject to BG optimization objectives, and thereby largely reduced LET_d_ in all critical OARs. TEopt strictly penalized track-ends and, thus, avoided elevated LET_d_ values to large volumes of the brainstem, while LETopt and DDopt focussed on reducing hotspots of track-ends and LET_d_. DRBEopt did not change the track-end distribution within the critical OARs substantially; instead, it produced a track-end hotspot at the border between CTV and brainstem to obtain an even steeper absorbed dose fall-off. Accordingly, brainstem LET_d_ values in DRBEopt remained comparable to DOSEopt (Fig. 5).

The observed D_RBE_ reductions by all BGopt approaches translated into significant average (range) NTCP(D_RBE_) reductions of 47.4 (33.3–63.9)% and were 2.6 (2.2–3.0) percentage points (pp), 9.2 (7.7–10.7) pp, 4.8 (3.7–5.3) pp and 5.2 (4.8–6.0) pp lower than DOSEopt for brainstem necrosis and blindness in the chiasm, right and left optic nerve, respectively. The highest NTCP(D_RBE_) reductions were observed for patients with elevated initial NTCP(D_RBE_) in the DOSEopt plans. All BGopt strategies performed equally well in terms of NTCP(D_RBE_) reductions and differences between BGopt strategies were 1.8 (0.5–6.2) pp and highest for DRBEopt in the high-dose group (Table 2).

**Table 2:** Normal tissue complication probabilities for dose-optimized plans and reductions achieved by biological effectiveness guided optimization

OAR, Endpoint	Group	Mean (range) NTCP(D_RBE_)/%	Mean (range) ΔNTCP(D_RBE_) to DOSEopt/percentage points			
		DOSEopt	TEopt	LETopt	DDopt	DRBEopt
Brainstem, necrosis						
	All	6.6 (0.7–18.0)	3.0* (0.5–8.6)	2.6* (0.4–8.0)	2.5* (0.6–6.0)	2.2* (0.2–7.0)
	D_pres_ ≤ 54	2.6 (0.7–4.8)	1.5 (0.5–2.3)	1.4 (0.5–2.3)	1.4 (0.6–2.1)	1.0 (0.2–2.2)
	D_pres_ > 54	10.6 (1.0–18.0)	4.5 (0.5–8.6)	3.8 (0.4–8.0)	3.6 (0.7–6.0)	3.5 (0.5–7.0)
Chiasm, blindness						
	All	16.8 (11.0–22.0)	8.8* (4.5–16.9)	7.7* (2.0–16.8)	9.5* (4.5–16.2)	10.7* (1.0–18.4)
	D_pres_ ≤ 54	16.3 (11.0–21.0)	6.1 (4.6–16.9)	6.1 (2.0–16.8)	5.7 (4.5–16.2)	7.1 (2.0–16.2)
	D_pres_ > 54	17.2 (12.0–22.0)	7.8 (4.5–10.7)	5.7 (2.3–8.0)	8.6 (4.9–10.9)	11.9 (1.0–18.4)
Opticus R, blindness						
	All	9.7 (0.2–20.0)	5.3* (0.2–9.0)	5.3* (0.2–10.4)	4.9* (0.2–10.0)	3.7* (0.1–16.5)
	D_pres_ ≤ 54	5.4 (0.2–9.3)	2.0 (0.2–3.0)	2.8 (0.2–6.1)	2.8 (0.2–5.4)	1.7 (0.1–3.0)
	D_pres_ > 54	13.0 (5.4–20.0)	6.2 (3.0–9.0)	5.6 (2.9–10.4)	6.4 (3.6–10.0)	9.2 (2.8–16.5)
Opticus L, blindness						
	All	11.2 (1.0–30.0)	5.2* (0.5–15.0)	4.8* (0.5–15.0)	4.9* (0.6–13.0)	6.0* (1.0–17.3)
	D_pres_ ≤ 54	13.5 (6.2–30.0)	6.5 (2.8–15.0)	6.6 (3.6–15.0)	6.4 (2.8–13.0)	5.7 (2.8–13.0)
	D_pres_ > 54	9.3 (1.0–27.0)	4.2 (0.5–12.0)	3.3 (0.5–8.0)	3.8 (0.6–9.0)	6.2 (1.0–17.3)

^*^p < 0.05 tested for group ‘all patients’, *D*_pres_ prescription dose, *NTCP* normal tissue complication probability, *D*_RBE_ variable RBE-weighted dose, *R* right, *L* left, *DOSEopt* dose-only optimization, *TEopt* track-end optimization, *LETopt* dose-averaged linear energy transfer (LET_d_) optimization with dose threshold, *DDopt* dirty dose optimization, *DRBEopt* variable RBE-weighted dose optimization

## Discussion

The amount of data on LET-driven RBE related side effects after proton therapy is increasing and originates predominantly from the analysis of brain toxicity. For brain irradiations, high patient positioning reproducibility, low initial beam energies and, thus, low range straggling, homogeneous anatomy and sharp distal absorbed dose gradients may contribute to high LET_d_ values and result in D_RBE_ uncertainties in normal tissue [39, 42]. Patients undergoing cranial proton therapy may particularly benefit from biologically guided treatment planning approaches beyond absorbed dose optimization since variable RBE-induced dose increase is primarily a spatially limited effect which may be particularly relevant for serial organs, as often found in cranial patients. Entities with parallel structured OARs are seldomly at a high initial risk of RBE-induced overdosages as the absorbed dose in proton therapy is typically far from OAR tolerance dose and thus, may limit the extend of a measurable benefit of BGopt.

The observed D_RBE_ reductions, using the Wedenberg model, by BG objectives translated into systematical NTCP(D_RBE_) reductions for brainstem necrosis and blindness. NTCP(D_RBE_) reductions by BGopt were about 50% with average and maximum reductions of 5.4 pp and 18.4 pp, respectively, compared to conventional DOSEopt plans (Table 2). BGopt strategies were particularly beneficial for patients with high initial risk in the DOSEopt plans. They achieved substantial NTCP reductions since absorbed dose values were already close to tolerance dose at which the sigmoid-shaped NTCP function changes rapidly with D_RBE_. The observed NTCP improvements in our study seem to reach a relevant level as they are in the same order of magnitude as NTCP differences used for patient selection to proton therapy by the model based approach [43] as adopted in countries such as the Netherlands and Denmark. It was found that none of the BG optimization strategies systematically outperformed the others in terms of NTCP(D_RBE_) reduction. Still, the absolute values for D_RBE_ and NTCP(D_RBE_) must be considered with caution [4], since the transferability of in-vitro data based RBE models to the clinical situation is limited, the applied NTCP models are based on photon data and the observed NTCP values for DOSEopt plans do not correlate to our clinical findings. The Wedenberg model with α/β = 2 Gy, as used in our study, incorporates the RBE dependencies known for protons from in-vitro data [3] and was found to be consistent with a clinically derived RBE model for brain tissue [8]. Additionally, different phenomenological RBE models share similar trends in variable RBE prediction for clinical scenarios and the same α/β [44], which reduces the impact of the choice of a particular RBE model in our study, while the absolute RBE and, thus, D_RBE_ values may be model-specific.

Relative to the DOSEopt reference plans, the average reduction in near-maximum LET_d_ to critical OARs was 19.0%, 19.0% and 18.9% with TEopt, LETopt and DDopt, respectively. This compares to earlier studies in brain tumor patients optimizing track-ends [28] or LET_d_ [24] showing an average LET_d_ reduction in near-maximum values of 25.7% and 23.7%, respectively. In contrast, the DRBEopt approach implemented here was found to be primarily absorbed dose-optimizing leaving the LET_d_ and RBE values on average largely unchanged. However, DRBEopt appeared less robust in the CTV compared to DOSEopt and the other BGopt plans when prescription doses were substantially above OAR tolerance dose. It is therefore desirable to implement BG planning strategies into a robust optimization framework to directly balance D_RBE_ reductions with maintaining plan robustness, especially when altering absorbed dose distributions. Robust optimization may impact the absorbed dose and LET_d_ distribution of the dose-only optimized PTV-based reference plan used in our study, particularly at the distal edge. Likewise, robust optimization may impact the effectiveness and benefit of all BGopt strategies which should be considered in future studies. The outcome of this study suggests that all BG plans should be complemented by robust evaluation when applied clinically.

For the BG optimization objectives, clinically relevant levels are required, first, to optimally balance D_RBE_ reductions with maintaining plan quality and, second, to systematically apply BGopt to a larger patient cohort prior to clinical translation. For LETopt, the applied levels were in accordance with clinically reported absorbed dose and LET_d_ values. Specifically, mean LET_d_ and mean D_1.1_ values above 2.5 keV/µm and 40 Gy(RBE), respectively, were reported within the anatomical brain regions that showed MR image changes after proton therapy [8, 12, 13, 29–31]. A more harmonized LET reporting in toxicity volumes would allow for deriving more consistent LET_d_-related volume objectives for plan optimization. Recently, the unrestricted LET_d_ of protons to water or unit density tissue was proposed [45].

DDopt presents a sensitive dose optimization approach as it allows for penalizing the absorbed dose contributions of high-LET protons. For DDopt and TEopt, however, neither voxelwise particle-energy spectra nor track-end fractions, respectively, have been reported so far for toxicity volumes. This hampers the derivation and application of suitable levels for DDopt and TEopt objectives and currently limits the exploitation of their full potential. Therefore, the LET_d_ values applied in LETopt were also used as LET threshold in DDopt. The maximum allowable track-end fraction falling in an OAR depends also on the incident field directions and the volume of the CTV and OAR [28]. Therefore, in our study, track-end levels to be penalized were found in trial-and-error iterations. A feasible advancement could be a track-end objective to minimize the track-ends in critical OARs without the use of a preset threshold. Although a 50% reduction in track-ends and near-maximum dirty dose kept plan quality and translated in NTCP(D_RBE_) reductions comparable to those in LETopt and DRBEopt, these objective levels may still not be optimal. Clearly, more studies reporting volume histogram parameters or voxel-wise information for absorbed dose, LET_d_ and dirty dose or track-ends in toxicity volumes are needed to better define the objectives to be used in biology-guided plan optimization strategies beyond absorbed dose optimization. Multi-criteria optimization with BGopt is required to quantify the impact of the uncertainties associated with the choice of user-specified threshold values on the resulting BGopt plans, as the impact also correlates with the applied objective weights.

DRBEopt may be the most intuitive optimization approach as it requires no change in treatment planning, except for replacing the current clinical RBE model, which assumes a constant value of 1.1, by a more appropriate and accepted variable clinical RBE model. Using tolerance doses from photon therapy as dose objectives for critical OARs appears safe since variable RBE typically exceeds 1.1 in critical OARs with low α/β. Consequently, the absorbed dose in these OARs decreases as was also observed in our study. However, DRBEopt in our current implementation decreased plan robustness in some cases in our study. Comparing the four considered BG optimization strategies, at this point in time, reducing LET_d_ in high-dose voxels with LETopt may therefore be favorable as it performed well in NTCP(D_RBE_) reductions, kept plan robustness and quality and objective levels were based on clinical toxicity reports.

BG optimization approaches can be applied for the target volume or OARs or for both. Furthermore, they may either optimize absorbed dose and dose-enhancing factors (track-ends, LET_d_, variable RBE) simultaneously (one-step) or sequentially (two-step). Here, a sequential approach was applied utilizing the clinically used treatment field angles for each patient. In a first step, constant RBE-weighted dose objectives were found that produced a clinically acceptable plan and remained fixed afterwards. Subsequently, dose-enhancing factors were added and optimized. This two-step approach generated favorable D_RBE_ to critical OARs and NTCP(D_RBE_) without degrading clinical plan quality, in line with similar studies [22, 26]. A simultaneous absorbed dose and LET_d_ optimization offers additional degrees of freedom and may reduce D_RBE_ and NTCP(D_RBE_) even further [17]. However, two-step optimization approaches are closer to current daily clinical treatment planning practice. Additionally, maintaining a constant RBE for dose prescription to the tumor is in line with recent recommendations and allows for consistent dose reporting while acquiring more clinical data on RBE variability [2]. Selectively reducing D_RBE_ to OARs is also in accordance with other research works on optimization of variable RBE related quantities in OARs [22, 23, 26, 30]. Therefore, sequentially reducing the LET_d_ to OARs while maintaining current dose constraints to the target volume is a first and safe step towards translating optimization with variable RBE into clinical practice.

## Conclusions

For the ten cranial patients in our study, the optimization of track-ends, LET_d_ in high-dose voxels, absorbed dose contributions by high-LET protons and variable RBE-weighted dose substantially reduced variable RBE-induced dose burden to healthy tissue. Simultaneously, all optimization strategies adhered to current dose reporting with a constant RBE in the target volumes. LET_d_ optimization in voxels with high absorbed dose may be favourable over the other strategies presented in this study, since optimization objectives can be based on clinical toxicity reports after cranial proton therapy and LET_d_-optimizing strategies were slightly superior in terms of robustness. Sequentially using biological effectiveness guided optimization strategies in OARs could be the first step with variable RBE into clinical practice while acquiring more clinical proton data on variable proton RBE.

## Data Availability

Research data are stored in an institutional repository and will be shared upon reasonable request to the corresponding author.

## Abbreviations

BG: Biological effectiveness guided
CI: Conformity index
CTV: Clinical target volume
DD: Dirty dose
D_RBE_: Variable relative biological effectiveness weighted dose
D_1.1_: Constant relative biological effectiveness weighted dose
Fx: Fractions
HI: Homogeneity index
LET: Linear energy transfer
LET_d_: Dose-averaged linear energy transfer
MC: Monte Carlo
MFO: Multi-field optimization
NTCP: Normal tissue complication probability
OAR: Organ at risk
Opt: Optimization
PTV: Planning target volume
RBE: Relative biological effectiveness
TE: Track-ends
TPS: Treatment planning system

## References

[1] Paganetti H, Niemierko A, Ancukiewicz M, Gerweck LE, Goitein M, Loeffler JS (2002). Relative biological effectiveness (RBE) values for proton beam therapy. Int J Radiat Oncol.

[2] Paganetti H, Blakely E, Carabe-Fernandez A, Carlson DJ, Das IJ, Dong L (2019). Report of the AAPM TG-256 on the relative biological effectiveness of proton beams in radiation therapy. Med Phys.

[3] Paganetti H (2014). Relative biological effectiveness (RBE) values for proton beam therapy. Variations as a function of biological endpoint, dose, and linear energy transfer. Phys Med Biol..

[4] Paganetti H (2022). Mechanisms and review of clinical evidence of variations in relative biological effectiveness in proton therapy. Int J Radiat Oncol.

[5] Peeler CR, Mirkovic D, Titt U, Blanchard P, Gunther JR, Mahajan A (2016). Clinical evidence of variable proton biological effectiveness in pediatric patients treated for ependymoma. Radiother Oncol.

[6] Underwood TSA, Grassberger C, Bass R, MacDonald SM, Meyersohn NM, Yeap BY (2018). Asymptomatic late-phase radiographic changes among chest-wall patients are associated with a proton RBE exceeding 1.1. Int J Radiat Oncol Biol Phys..

[7] Eulitz J, Troost EGC, Raschke F, Schulz E, Lutz B, Dutz A (2019). Predicting late magnetic resonance image changes in glioma patients after proton therapy. Acta Oncol.

[8] Bahn E, Bauer J, Harrabi S, Herfarth K, Debus J, Alber M (2020). Late contrast enhancing brain lesions in proton-treated patients with low-grade glioma: clinical evidence for increased periventricular sensitivity and variable RBE. Int J Radiat Oncol.

[9] Wang C-C, McNamara AL, Shin J, Schuemann J, Grassberger C, Taghian AG (2020). End-of-range radiobiological effect on rib fractures in patients receiving proton therapy for breast cancer. Int J Radiat Oncol Biol Phys.

[10] Engeseth GM, He R, Mirkovic D, Yepes P, Mohamed ASR, Stieb S (2021). Mixed effect modeling of dose and linear energy transfer correlations with brain image changes after intensity modulated proton therapy for skull base head and neck cancer. Int J Radiat Oncol Biol Phys.

[11] Harrabi SB, von Nettelbladt B, Gudden C, Adeberg S, Seidensaal K, Bauer J (2022). Radiation induced contrast enhancement after proton beam therapy in patients with low grade glioma: How safe are protons?. Radiother Oncol.

[12] Niemierko A, Schuemann J, Niyazi M, Giantsoudi D, Maquilan G, Shih HA (2021). Brain necrosis in adult patients after proton therapy: is there evidence for dependency on linear energy transfer?. Int J Radiat Oncol.

[13] Garbacz M, Cordoni FG, Durante M, Gajewski J, Kisielewicz K, Krah N (2021). Study of relationship between dose, LET and the risk of brain necrosis after proton therapy for skull base tumors. Radiother Oncol.

[14] Sørensen BS, Pawelke J, Bauer J, Burnet NG, Dasu A, Høyer M (2021). Does the uncertainty in relative biological effectiveness affect patient treatment in proton therapy?. Radiother Oncol.

[15] Heuchel L, Hahn C, Pawelke J, Sørensen BS, Dosanjh M, Lühr A (2022). Clinical use and future requirements of relative biological effectiveness: Survey among all European proton therapy centres. Radiother Oncol.

[16] Haas-Kogan D, Indelicato D, Paganetti H, Esiashvili N, Mahajan A, Yock T (2018). National cancer institute workshop on proton therapy for children: considerations regarding brainstem injury. Int J Radiat Oncol.

[17] Deng W, Yang Y, Liu C, Bues M, Mohan R, Wong WW (2021). A critical review of LET-based intensity-modulated proton therapy plan evaluation and optimization for head and neck cancer management. Int J Part Ther.

[18] Tommasino F, Durante M (2015). Proton radiobiology. Cancers.

[19] Bauer J, Bahn E, Harrabi S, Herfarth K, Debus J, Alber M (2021). How can scanned proton beam treatment planning for low-grade glioma cope with increased distal RBE and locally increased radiosensitivity for late MR-detected brain lesions?. Med Phys.

[20] Grassberger C, Trofimov A, Lomax A, Paganetti H (2011). Variations in linear energy transfer within clinical proton therapy fields and the potential for biological treatment planning. Int J Radiat Oncol Biol Phys.

[21] Giantsoudi D, Grassberger C, Craft D, Niemierko A, Trofimov A, Paganetti H (2013). Linear energy transfer-guided optimization in intensity modulated proton therapy: feasibility study and clinical potential. Int J Radiat Oncol Biol Phys.

[22] Unkelbach J, Botas P, Giantsoudi D, Gorissen BL, Paganetti H (2016). Reoptimization of intensity modulated proton therapy plans based on linear energy transfer. Int J Radiat Oncol.

[23] An Y, Shan J, Patel SH, Wong W, Schild SE, Ding X (2017). Robust intensity-modulated proton therapy to reduce high linear energy transfer in organs at risk. Med Phys.

[24] Cao W, Khabazian A, Yepes PP, Lim G, Poenisch F, Grosshans DR (2017). Linear energy transfer incorporated intensity modulated proton therapy optimization. Phys Med Biol.

[25] Liu C, Patel SH, Shan J, Schild SE, Vargas CE, Wong WW (2020). Robust optimization for intensity modulated proton therapy to redistribute high linear energy transfer from nearby critical organs to tumors in head and neck cancer. Int J Radiat Oncol.

[26] Sánchez-Parcerisa D, López-Aguirre M, Dolcet Llerena A, Udías JM (2019). MultiRBE: treatment planning for protons with selective radiobiological effectiveness. Med Phys.

[27] Henjum H, Dahle TJ, Fjæra LF, Rørvik E, Pilskog S, Stokkevåg CH (2021). The organ sparing potential of different biological optimization strategies in proton therapy. Adv Radiat Oncol.

[28] Traneus E, Ödén J (2019). Introducing proton track-end objectives in intensity modulated proton therapy optimization to reduce linear energy transfer and relative biological effectiveness in critical structures. Int J Radiat Oncol Biol Phys.

[29] Giantsoudi D, Sethi RV, Yeap BY, Eaton BR, Ebb DH, Caruso PA (2016). Incidence of CNS injury for a cohort of 111 patients treated with proton therapy for medulloblastoma: LET and RBE associations for areas of injury. Int J Radiat Oncol Biol Phys.

[30] Ödén J, Toma-Dasu I, Witt Nyström P, Traneus E, Dasu A (2020). Spatial correlation of linear energy transfer and relative biological effectiveness with suspected treatment-related toxicities following proton therapy for intracranial tumors. Med Phys.

[31] Bertolet A, Abolfath R, Carlson DJ, Lustig RA, Hill-Kayser C, Alonso-Basanta M (2022). Correlation of LET with MRI changes in brain and potential implications for normal tissue complication probability for patients with meningioma treated with pencil beam scanning proton therapy. Int J Radiat Oncol.

[32] Cortés-Giraldo MA, Carabe A (2015). A critical study of different Monte Carlo scoring methods of dose average linear-energy-transfer maps calculated in voxelized geometries irradiated with clinical proton beams. Phys Med Biol.

[33] Wedenberg M, Lind BK, Hårdemark B (2013). A model for the relative biological effectiveness of protons: the tissue specific parameter α/β of photons is a predictor for the sensitivity to LET changes. Acta Oncol.

[34] Mayo C, Yorke E, Merchant TE (2010). Radiation associated brainstem injury. Int J Radiat Oncol.

[35] Mayo C, Martel MK, Marks LB, Flickinger J, Nam J, Kirkpatrick J (2010). Radiation dose-volume effects of optic nerves and chiasm. Int J Radiat Oncol.

[36] Lambrecht M, Eekers DBP, Alapetite C, Burnet NG, Calugaru V, Coremans IEM (2018). Radiation dose constraints for organs at risk in neuro-oncology; the European Particle Therapy Network consensus. Radiother Oncol.

[37] Bentzen SM, Constine LS, Deasy JO, Eisbruch A, Jackson A, Marks LB (2010). Quantitative analyses of normal tissue effects in the clinic (QUANTEC): an introduction to the scientific issues. Int J Radiat Oncol Biol Phys.

[38] Lowe M, Albertini F, Aitkenhead A, Lomax AJ, MacKay RI (2016). Incorporating the effect of fractionation in the evaluation of proton plan robustness to setup errors. Phys Med Biol.

[39] Hahn C, Eulitz J, Peters N, Wohlfahrt P, Enghardt W, Richter C (2020). Impact of range uncertainty on clinical distributions of linear energy transfer and biological effectiveness in proton therapy. Med Phys.

[40] Källman P, Ågren A, Brahme A (1992). Tumour and normal tissue responses to fractionated non-uniform dose delivery. Int J Radiat Biol.

[41] Ågren Cronqvist A-K (1995). Quantification of the response of heterogeneous tumours and organized normal tissues to fractionated radiotherapy.

[42] Grün R, Friedrich T, Krämer M, Zink K, Durante M, Engenhart-Cabillic R (2013). Physical and biological factors determining the effective proton range. Med Phys.

[43] Langendijk JA, Lambin P, De Ruysscher D, Widder J, Bos M, Verheij M (2013). Selection of patients for radiotherapy with protons aiming at reduction of side effects: The model-based approach. Radiother Oncol.

[44] McMahon SJ (2021). Proton RBE models: commonalities and differences. Phys Med Biol..

[45] Hahn C, Ödén J, Dasu A, Vestergaard A, Fuglsang Jensen M, Sokol O (2022). Towards harmonizing clinical linear energy transfer (LET) reporting in proton radiotherapy: a European multi-centric study. Acta Oncol.

